# Bayesian hypothesis testing and experimental design for two-photon imaging data

**DOI:** 10.1371/journal.pcbi.1007205

**Published:** 2019-08-02

**Authors:** Luke E. Rogerson, Zhijian Zhao, Katrin Franke, Thomas Euler, Philipp Berens

**Affiliations:** 1 Institute for Ophthalmic Research, University of Tübingen, Tübingen, Germany; 2 Center for Integrative Neuroscience, University of Tübingen, Tübingen, Germany; 3 Bernstein Center for Computational Neuroscience, University of Tübingen, Tübingen, Germany; 4 Graduate Training Center for Neuroscience, University of Tübingen, Tübingen, Germany; 5 Institute for Bioinformatics and Medical Informatics, University of Tübingen, Tübingen, Germany; Stony Brook University, UNITED STATES

## Abstract

Variability, stochastic or otherwise, is a central feature of neural activity. Yet the means by which estimates of variation and uncertainty are derived from noisy observations of neural activity is often heuristic, with more weight given to numerical convenience than statistical rigour. For two-photon imaging data, composed of fundamentally probabilistic streams of photon detections, the problem is particularly acute. Here, we present a statistical pipeline for the inference and analysis of neural activity using Gaussian Process regression, applied to two-photon recordings of light-driven activity in *ex vivo* mouse retina. We demonstrate the flexibility and extensibility of these models, considering cases with non-stationary statistics, driven by complex parametric stimuli, in signal discrimination, hierarchical clustering and other inference tasks. Sparse approximation methods allow these models to be fitted rapidly, permitting them to actively guide the design of light stimulation in the midst of ongoing two-photon experiments.

## Author summary

There are many sources of noise in recordings of neural activity, and the first challenge in neural data analysis is to separate this noise from experimentally relevant variation. This is particularly problematic for two-photon imaging data. Two-photon imaging uses fluorescent indicators to measure changes in the concentration of molecules involved in cell signalling, and adds a variety of numerical, biological and optical noise sources. We present a method for disentangling this signal and noise using Gaussian processes, a family of probabilistic models which provide a principled way of inferring mean activity and variability. In addition to signal recovery, we show that these models can test the evidence for whether and where two signals are different and that these tests can be used to look for groups in sets of signals. We explore how these models can be extended to predict how signals will change under different experimental conditions, and that these predictions can be used to select new conditions for further exploration.

## Introduction

Over the last two decades, two-photon (2P) imaging has become one of the premier tools for studying coding in neural systems from the population level down to individual neural compartments [1]. The resulting data is highly variable due to the inherent variability of neurons and technical sources of noise in the imaging process [2, 3]. Yet we typically assume that beneath the noisy signals which are observed there is a smooth latent function describing the activity of a neuron or a neural compartment. In a typical analysis pipeline for 2P data, we attempt to recover this function by grouping noisy observations from pixels into regions of interest (ROIs), which cover the soma or different compartments of a neuron, temporally interpolating them to a common frame rate and averaging across repetitions of the same stimulus (see also Box 1). Each stage is intended to smooth the observations and get closer to the “true” underlying activity function of the neuron. To measure the uncertainty about this latent activity function, often the variance between repetitions of the same experimental condition is used, with little assessment of whether this reflects the actual uncertainty given measurement and neural variability.

**Box 1: Analysis pipelines for two-photon imaging data**

Two-photon imaging data can typically be described by two spatial dimensions (*x*, *y*) and a time dimension (*t*), at which fluorescence of an activity indicator is measured. In addition, there is a second set of variables one needs to consider: those of the stimulus, which are described with a set of parameters (*θ*). Thus, a single measurement *F*_*x*,*y*,*t*,*θ*_ describes the fluorescence at point (*x*, *y*) and time *t*, given that stimulus *θ* was shown. The dimensionality of the parameter vector will vary between stimuli and experiments.

In an analysis pipeline, we want to perform inference about e.g. whether a stimulus parameter systematically influences the neural activity, i.e. whether $F_{x,y,t,\theta_{1}}=F_{x,y,t,\theta_{2}}$. The first step of such a pipeline is often to transform the fluorescence data *F*_*x*,*y*,*t*,*θ*_ into time-series representations for a distinct neural structure such as the soma or an axon. This transformation is usually achieved by defining a set of regions of interest (ROI) in the spatial dimensions and for each of those, computing the average value of all measured values in each time point:

$$F_{i,t,\theta }={\left\langle F_{x,y,t,\theta }\right\rangle }_{x,y\in ROI_{i}}$$ (1)

Here, 〈〉 denote the average. Two-photon imaging provides only near-simultaneous measures of the structures in the imaging field, as the scanning laser which transverses the field is only positioned at one point within the field at any given time. At no time point are two structures truly simultaneously recorded. The activity of each structure at a common set of time points therefore needs to be inferred to enable the averaging procedure.

The classical pipeline solves the above issues as follows: ROIs are specified, the state of the ROI is inferred from the mean of its pixel (x,y) values in each scan frame, and interpolation (e.g. cubic spline) is used to infer the activity of the ROI at a common set of time points. Signal uncertainty is calculated by computing the variance of the interpolated signals between stimulus trials. The stimulus parameters are not explicitly modelled in this pipeline.

In our proposed pipeline, the mean value of the activity of each ROI is computed for each scan line where this ROI is scanned (corresponding to either the horizontal line in a linear scan, or one complete arc in the spiral scan), and a Gaussian Process is used to infer the activity of the ROIs over time and over the stimulus parameters. The variance of these signals is directly inferred as part of the Gaussian process model, decomposed into additive observation noise and uncertainty associated with the mean signal (referred to as “latent uncertainty” throughout, compare Box 2).

Here, we propose a different approach based on Gaussian Process (GP) regression [4] to infer signals from 2P recordings in a statistically principled manner, propagating the uncertainty all the way from the measurements to the desired inference. This regression procedure recovers an estimate of the true activity of the neuron, whether changes in calcium or glutamate concentration, from observations with experimental noise. This is facilitated by modelling explicitly the change in the signal over time and as a function of stimulus parameters. Gaussian processes are probabilistic models, which describe the functional relationship between a set of predictors and a set of observations (see Box 2 for a mathematical primer). In contrast to typical pre-processing pipelines, the statistical properties of the observed signal are considered explicitly as part of the model optimisation. Recently developed sparse GP approximations allow us to apply these models to comparatively large datasets with several thousand observations, as are common in 2P experiments [5].

**Box 2: Mathematics of Gaussian Process regression**

Gaussian processes are distributions over functions, with a mean function and a covariance kernel (for further mathematical details, see [4]):

$$y=f\left(X\right)∼GP(m\left(X\right),k\left(X,X^{\prime }\right))$$ (2)

m(X)=E[f(X)] (3)

$$k(X,X^{\prime })=E[\left(f\left(X\right)-E\left[f\left(X\right)\right]\right)\left(f\left(X^{\prime }\right)-E\left[f\left(X^{\prime }\right)\right]\right)]$$ (4)

The covariance kernel models how the function varies as the predictors change. Many common covariance kernels including the Radial Basis Function and Exponential kernels are members of the class of Matérn covariance kernels. We use the RBF kernel throughout our pipeline. The kernel’s length-scale *l* hyperparameter models the rate at which the observations are expected to change as a function of the predictors.

$$k_{RBF,\phi }\left(X,X^{\prime }\right)=\sigma_{signal}^{2}\text{exp}\left(-\frac{∥X-X^{\prime }{∥}^{2}}{2l^{2}}\right)+I\sigma_{noise}^{2}$$ (5)

$$\phi =\{l,\sigma_{signal},\sigma_{noise}\}$$ (6)

Here *I* is the identity matrix. The signal variance *σ*_*signal*_ models the amplitude of the signal. The noise variance *σ*_*noise*_ reflects the uncertainty associated with the signal, and is modelled as additive Gaussian distributed noise. Jointly, the kernel and its hyperparameters form a prior distribution over the function we wish to infer. We used the log marginal likelihood

$$\begin{array}{c} log\left(p\left(y|X\right)\right)=-\frac{1}{2}y^{T}{\left[k\left(X,X^{\prime }\right)\right]}^{-1}y\\ -\frac{1}{2}log\left|\left[k\left(X,X^{\prime }\right)\right]\right|+-\frac{n}{2}log2\pi \end{array}$$ (7)

as an objective function to define the quality of the model with respect to the observations and to infer the hyperparameters *ϕ*. In the simplest case of Fig 2, where we model the activity of ROI *i* to local or full-field chirp stimuli described by the parameter vector *θ*, the predictor matrix *X* = (*t*_1_, *t*_2_, …, *t*_*T*_) simply corresponds to the time points and the neural activity is modeled as

$$F_{i,\theta }∼GP\left(m\left(X\right),k_{RBF,\phi }\left(X,X^{\prime }\right)\right)$$ (8)

The inferred function, where the GP has been conditioned on the observations, inferred for a new set of points, is defined as

$$\begin{array}{c} F_{i,\theta }^{*}|X^{*},X,F_{i,\theta }\\ ∼N(\mu_{\phi }(X^{*},X),\\ \Sigma_{\phi }\left(X^{*},X\right)) \end{array}$$ (9)

The stimulus *θ* determines the predictor matrices *X* and X*, which can include more stimulus parameters than time (sinusoid, moving bar). The functions *μ*_*p*_*hi* and Σ_*ϕ*_ are defined in Materials and methods. The hyperparameters *σ*_*signal*_ and *σμ*_*ϕ*_ can be used to compute uncertainty estimates of the neural activity. The uncertainty associated with the mean function, which we refer to as the “latent uncertainty”, is calculated using Eq (9), where the Gaussian noise component $\sigma_{noise}^{2}$ is excluded in the kernel *k* (see Materials and methods).

Using 2P recordings of calcium and glutamate dynamics in isolated mouse retina, we demonstrate how these models can be used to construct probabilistic representations of neural activity. We treat several use cases: First, we show that GP-based analysis of 2P recordings can be used to perform comparisons between the responses of a given cell under different conditions, allowing one to identify parts of the response with significant differences. Second, we exploit the properties of the GPs to perform a hierarchical clustering of cell responses and provides quantitative criteria for deciding how many clusters to keep. In addition, we use the framework to test which stimulus parameters influence neural activity in an ANOVA-like framework. Finally, we explore how the representation of uncertainty can be exploited for experimental design, informing the choice of parameters to optimally reduce the uncertainty about the neural response.

## Results

We applied a Bayesian framework based on Gaussian Process (GP) regression to efficiently infer neural activity with uncertainty estimates from recordings of light stimulus-driven activity in the mouse retina. The retina decomposes a stream of images into parallel channels representing salient stimulus features. The central circuit of this network is a feedforward pathway relaying the initial signal from the photoreceptors through the intermediate bipolar cells to retinal ganglion cells (RGCs), and from there through the optic nerve to the rest of the visual system. Inhibitory interneurons called horizontal and amacrine cells play key roles in the adaptation and feature extraction (for review, see [6]). In the datasets analysed here, we measured three stages of the excitatory pathway: Firstly, the presynaptic calcium signal in the axon terminals of a bipolar cell using the synthetic indicator dyes Oregon-Green BAPTA-1 (OGB-1) and GCaMP6f (the latter data previously published in [7]). Secondly, the glutamate release from these terminals, as measured by the genetically-encoded biosensor iGluSnFR [8, 9]. Finally, the calcium signal in RGC somata loaded with OGB-1 through bulk electroporation [10].

In our framework, a GP model (see Box 2 for mathematical primer) infers an estimate of the activity of a neuron from the observed fluorescence of each ROI in a scan field, which are typically cell somata or axon terminals. The estimated function models the “true” activation state of the neuron, i.e. the concentration of calcium or glutamate. In addition, we model the uncertainty about the estimated function including the observation noise, and the latent uncertainty about the activity function, once the observation noise is removed.

### Modelling uncertainty using Gaussian processes

**Fig 1:**
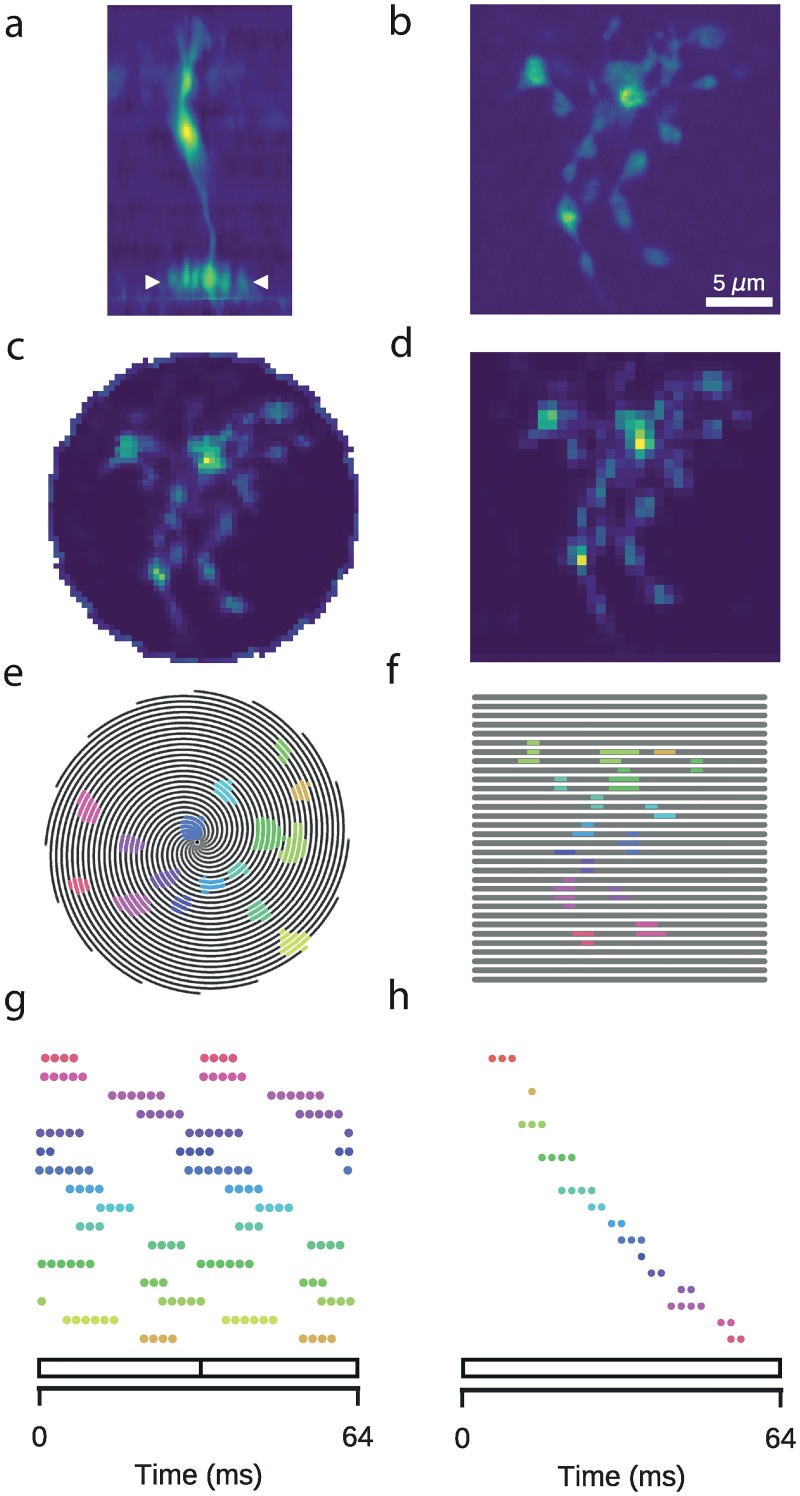
Two-photon imaging of retinal neurons. Data: Retinal bipolar cell filled with OGB-1 via sharp electrode injection and recorded using linear and spiral scan configurations. **a**: Vertical profile. Image coloured according to fluorescence intensity (yellow: high, blue: low). **b**: Horizontal (x-y), high-resolution scan of axon terminal system (512 x 512 pixels), corresponding to the domain between the two white ticks in (a). **c**: Spiral scan of axon terminal system (16 spirals; 31.25 Hz), as above. **d**: Linear scan of axon terminal system (32 lines; 15.625 Hz), as above. **e**: Spiral scan trajectory with ROI mask superimposed. Black lines indicate scan trajectory. Colours correspond to discrete ROIs. **f**: As (e), but with a linear scan configuration. The same ROIs were used. **g**: Time points at which the scan trajectory intersects with the ROI mask in (e). 64 ms span corresponds to two spiral scan frames. **h**: As (g), but for a linear scan configuration. ROIs correspond to those in (f). 64 ms span corresponds to one linear scan frame.

Our first objective was to infer the neural activity function and its associated uncertainty from our observations of the activity of single ROIs, located on individual synaptic axon terminals of a bipolar cell. In bipolar cells injected with the calcium indicator OGB-1, it is possible to image the anatomy of the cell by recording 3D stacks of x-y images at regular intervals along the vertical (z) axis (Fig 1a). High resolution scans allowed us to identify individual axon terminals (Fig 1b). Faster scans with lower spatial resolution are required to resolve neural activity, although the required reduction in resolution is substantially less for spiral configurations relative to classical linear configurations (Fig 1c and 1d). Although the scan patterns are highly regular, the spatial organisation of the neural structures results in irregular sampling over time (Fig 1e–1h).

**Fig 2:**
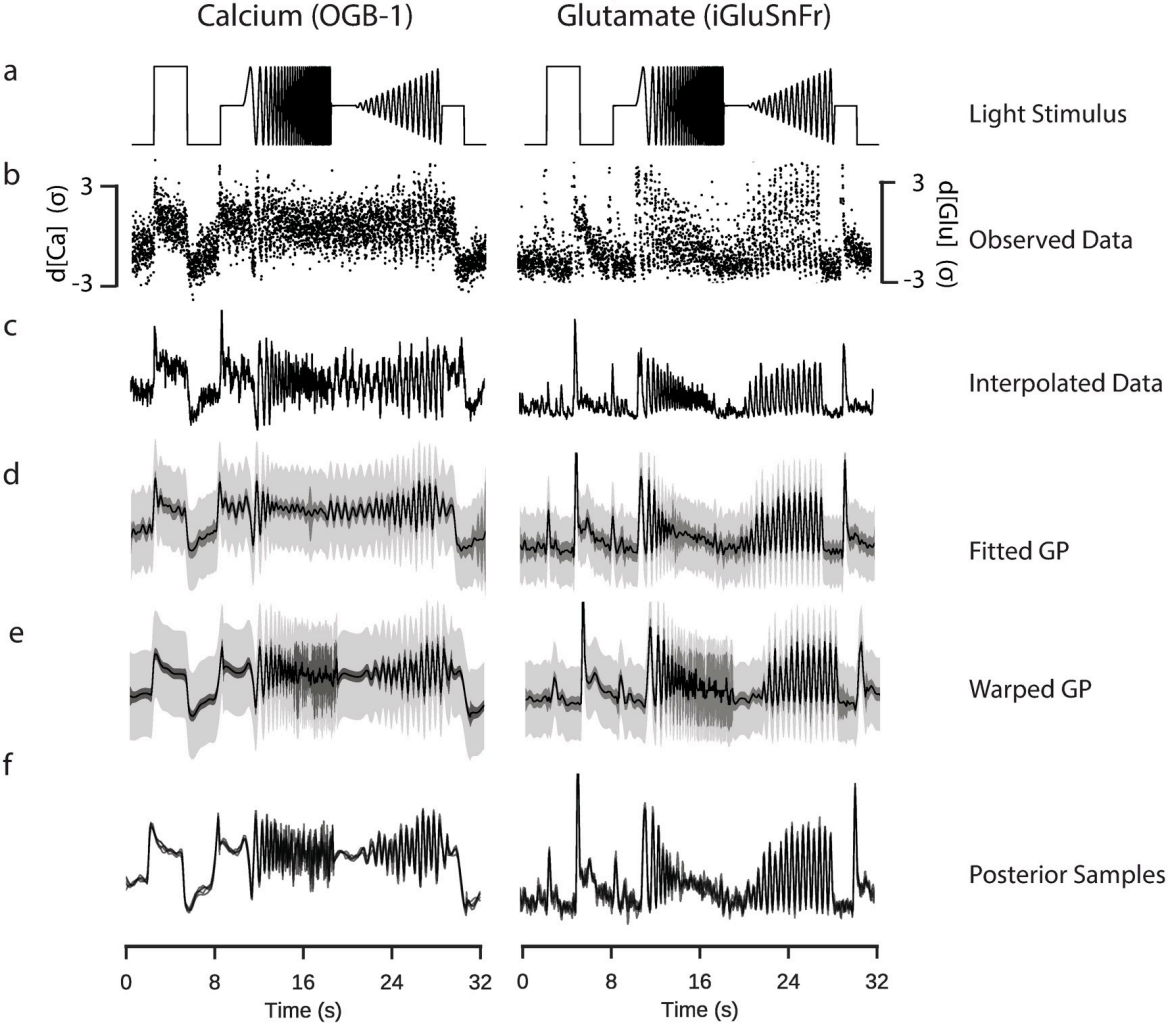
Inference of signals from two-photon data. Data: ROI from a retinal bipolar cell filled with OGB-1 via sharp electrode injection (left), and a different ROI from a scan field with bipolar cell terminals in a retina expressing iGluSnFR (right); both recorded using spiral scan configurations. Model: RBF kernel, 300 inducing inputs, 25 iterations per fit, best of 6 fits per model. **a**: “Full-field chirp” light stimulus, consisting of a light step, a frequency-modulated sine wave and a contrast-modulated sine wave. **b**: Observed activity of a single ROI. Each point corresponds to the mean activity of the ROI in a single scan line. The time at which the point was recorded is defined relative to the start of each stimulus trial, such that each trial leads to at least one data point for every time the laser scans across a given ROI. Information regarding the trial from which the point was derived is not explicitly incorporated into the model. **c**: Estimate of underlying signal from frame averaging, cubic spline interpolation and averaging over trials. This corresponds to the typical approach used in previous papers [7]. **d**: Fitted sparse Gaussian process. Black line indicates the mean signal. Intervals indicate uncertainty of the signal with and without the observation noise (light and dark grey, respectively), to 3 standard deviations. **e**: Fitted sparse warped Gaussian process. Input warping uses the warping function shown in the following figure. Model has been projected back onto the original time dimension. **f**: Five posterior samples drawn from the fitted sparse warped Gaussian process models.

We recorded bipolar cell calcium and glutamate signals measured during the presentation of a spatially homogeneous light stimulus including a light step and variations in temporal frequency and contrast (Fig 2a, chirp stimulus), as used in previous studies [7, 11]. We used the observed activity of a ROI (Fig 2b), and inferred a signal for each repeat using frame-averaging and cubic-spline interpolation (Fig 2c), corresponding to the classical way of inferring these functions (i.e. [7, 11]).

We then fitted a GP with a radial basis function (RBF) kernel for the time dimension to the observed activity (Fig 2d). We monitored the computation time and calculated the likelihood of an out-of-sample test set to determine a suitable number of inducing inputs. Surprisingly, this indicated that there was already little improvement in the performance of the model when more than 250 data points were used (S1a and S1b Fig), and that relatively few iterations of the fitting algorithm were required (S1c Fig).

**Fig 3:**
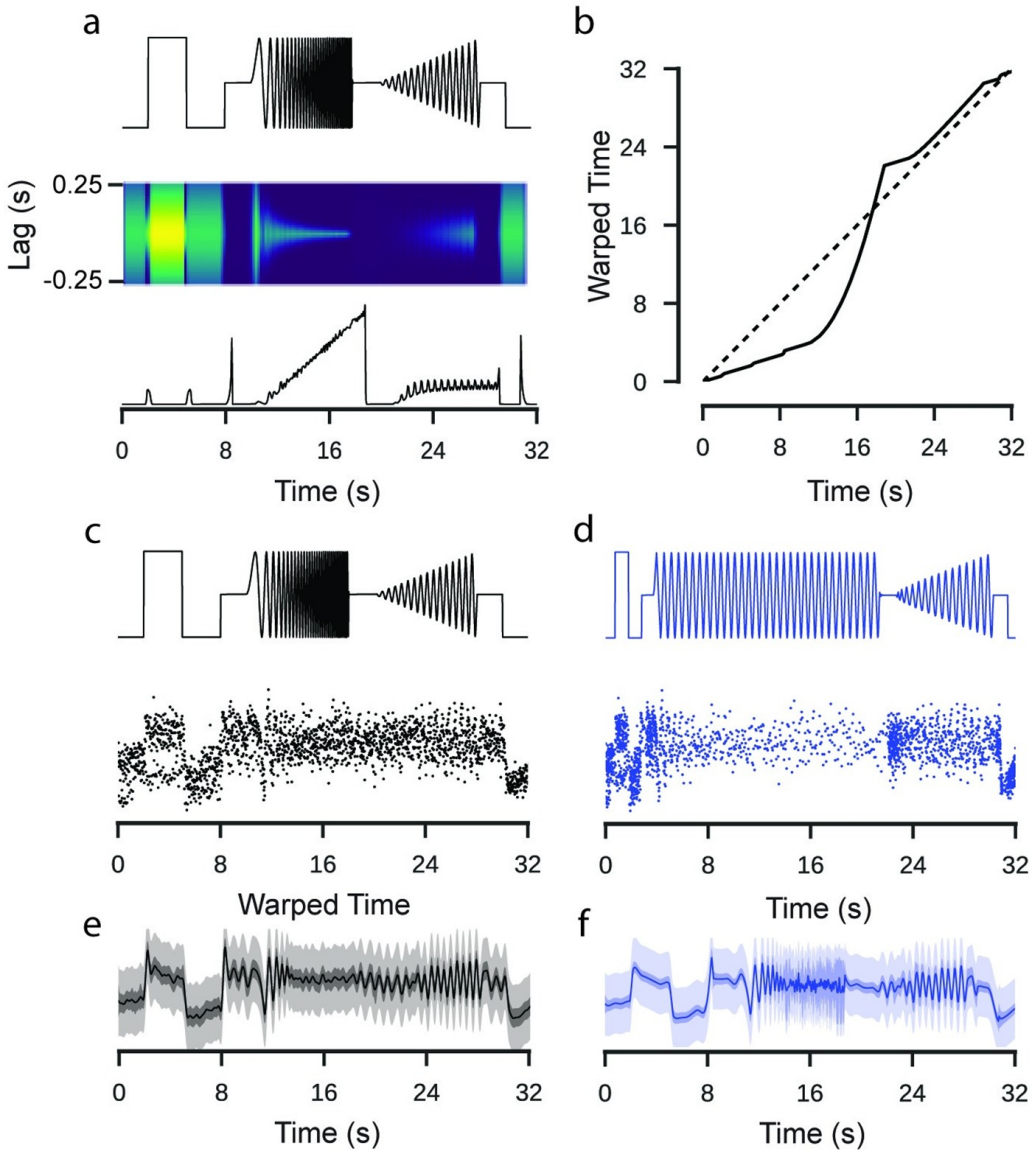
Application of a warping function to model input features. Data: ROI from a retinal bipolar cell filled with OGB-1 via sharp electrode injection. Model: RBF kernel, 300 inducing inputs, 20 iterations per fit, best of 3 fits per model. **a**: “Full-field chirp” stimulus (top). Autocorrelation functions corresponding to Gaussian curves fit to the empirical autocorrelation function over a 500 ms window (middle). Length scale parameter of the Gaussian distribution fitted to the autocorrelation functions. **b**: Cumulative sum of the inverse lengthscale over time. If the signal were stationary, the lengthscale would be constant, corresponding to the dashed line. This cumulative sum maps time onto a warped time dimension. **c**: Full field chirp stimulus with observations of the activity of one ROI labelled with OGB-1. **d**: The same stimulus and observations after a warping operation has been applied. **e**: GP fitted to the original data. **f**: GP fitted to the warped data. The function has been projected back onto un-warped time. Note the increased uncertainty in regions where the stimulus is changing rapidly and the variations in the smoothness of the inferred signal over time.

To account for temporal non-stationarities in the neural response, we then compared the GP model to an extended model with input warping (see Methods). One assumption of classical GP models is that the function space has a stationary autocorrelation function, i.e. that its correlational structure does not change with respect to a predictor, such as time. However, light induced neural activity like responses to the chirp, which have highly non-stationary correlational structure, are likely to show a commensurate non-stationarity in the response. We computed a warping function which transforms the time dimension such that the stimulus input has a stationary autocorrelation structure (Fig 3a). We then used this warping function to transform the input to the GP model of the response, under the assumption that the correlational structure of the response matched that of the stimulus input [12, 13](Fig 3b–3d). By performing this extra processing step, we were able to fit a model which could vary in its autocorrelation.

Our results show a clear difference between the predictions of the warped GP model and the simpler stationary one. In the simpler model, the selected parameters reflect a trade-off between models which fit closely to each of the different stimulus components (i.e. steps vs. chirps), resulting in an inferred mean signal which appears noisy during the light step and poorly tracks the faster chirp oscillations. As a consequence, the inferred uncertainty was relatively stationary over time (Fig 3e). By contrast, in the warped GP model, the inferred mean signal during the light step was smoother and tracked the faster oscillations much more closely (Fig 3f). More importantly, in contrast to the interpolated signal derived by a classical pipeline, the warped GP infers a high level of uncertainty during periods of rapid oscillation which are at, or close to, the sampling limit of the recording.

In practice, we found that the approach described above was more stable and faster than inferring the autocorrelation function directly from the observed activity. This appeared to be due to two factors: the irregular sampling distribution of the observed activity and the observation noise. Estimating the autocorrelation function separately for each ROI added a considerable computational burden to the pre-processing pipeline. In principle, the approach demonstrated can be applied to any stimulus with a known time-course if it is spatially homogeneous. Where this is not the case, the temporal statistics of the observed response may also be influenced by spatial integration and an alternative model, which explicitly accounted for this, would be appropriate.

### Using GP models for statistical inference

The key benefit of the GP framework is that it provides an explicit estimate of the uncertainty about the neural activity which can be used to perform well calibrated statistical inference, e.g. for inferring which periods of neural activity differed between two conditions. This is in contrast to classical approaches, where typical analysis follows multiple smoothing steps and often only the inter-trial variability is considered, providing a poorly calibrated estimate of uncertainty.

**Fig 4:**
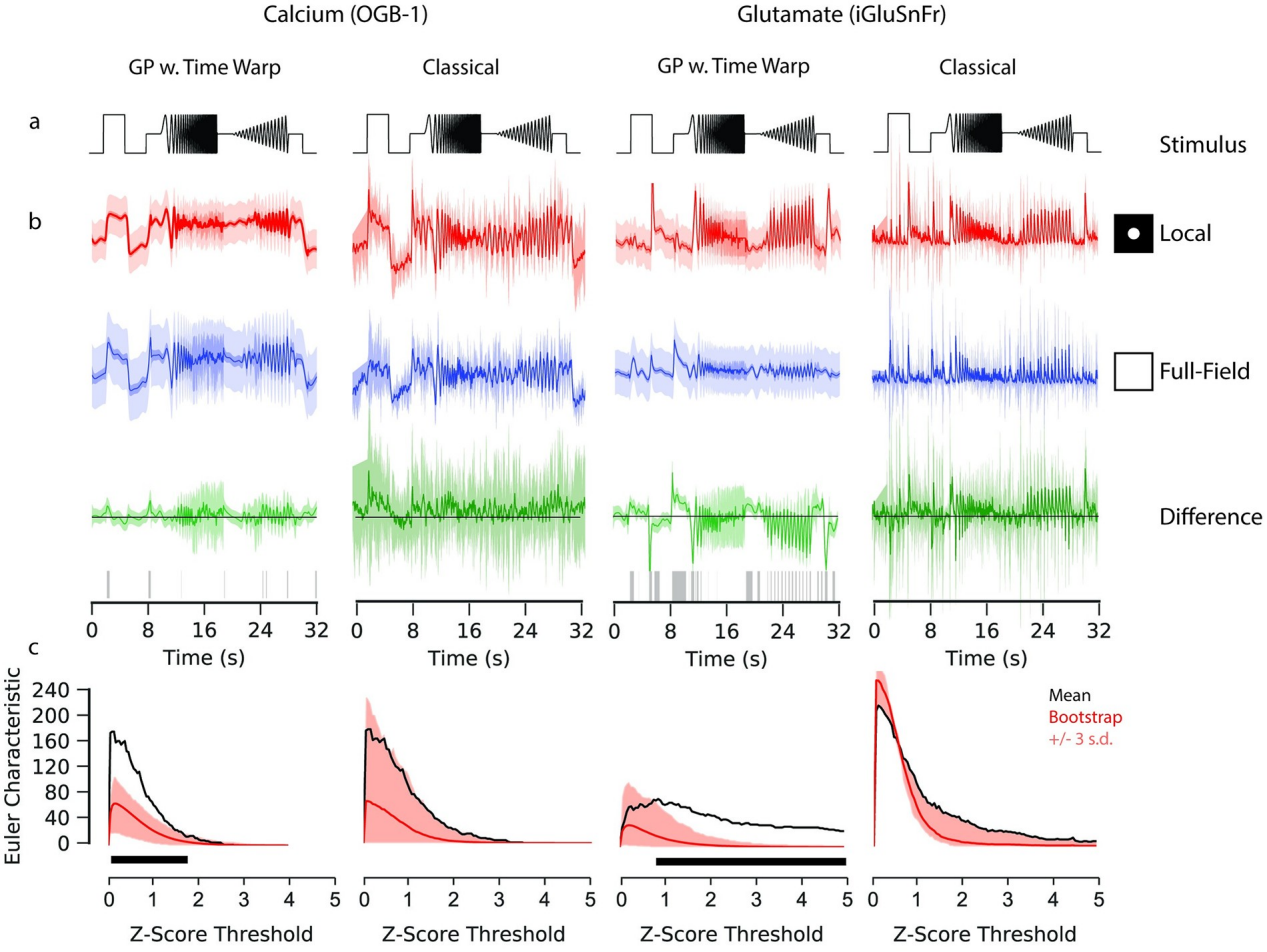
Gaussian process equality testing. Data: ROI from a retinal bipolar cell filled with OGB-1 by sharp electrode injection (left), and a different ROI from a scan field with bipolar cell terminals in a retina expressing iGluSnFR (right); both recorded using spiral scan configurations. Model: GP w. Time Warp: RBF kernel, 300 inducing inputs, 20 iterations per fit, best of 5 fits per model. Classical: pipeline incorporating frame averaging and interpolation. **a**: “Chirp” light stimulus. **b**: Fits of the GP with time warping and classical pipeline to chirp-driven responses, for calcium and glutamate data. Models fitted to observations of the responses to local (100 *μ*m; top) and full field (middle) chirp stimulus. Circles indicate relative spatial extent of the light stimulus. Difference between the models for the two stimulus conditions shown at the bottom. Intervals for the response data show 3 standard deviations above and below the mean function. This variability corresponds to the standard deviation with and without additive noise for the GPs, and the inter-trial standard deviation for the classical pipeline. Only the standard deviation without additive noise is shown for the difference of the GPs. Domains where zero-vector not included within this interval are highlighted with grey ticks, corresponding to regions where the difference between the two signals is greater than expected by chance. **c**: Frequency of discrete domains where zero-vector is not included in the credible intervals (also known as the Euler Characteristic; EC) as a function of the number of standard deviations above and below the respective mean functions. A high EC indicates a large degree of statistical separation between the two signals, and typically declines as the threshold increases. Bootstrap estimates of the null distribution of the EC are superimposed, with the mean of the null distributed shown in red. Intervals correspond to three standard deviations above and below the mean of the null distribution. The black box indicates the thresholds where the EC from the difference test exceeds the highest estimate from the null distribution by three standard deviations.

In our framework, we use a GP equality test to identify whether two signals are statistically different [14]. As an example, we consider the response of bipolar cells to the chirp stimulus as a function of the spatial extent of the light spot. This is known to modulate bipolar cell responses, with the difference being induced by lateral inhibition [7, 15–17]. We compared the calcium and glutamate signals of bipolar cells presented with chirp stimuli whose light spots differed in size (100*μm* and full field). We fitted a GP model with time warping to each of the sets of observations (Fig 4a and 4b), performed maximum likelihood estimation to optimise the GP parameters with respect to the data, and then computed the difference between the estimated latent functions.

We identified the periods of activity where the stimulus drives greater differences in the response than would be expected by chance (defined as the three standard deviations around the estimated difference function not including zero). We found the number of disconnected regions where the difference is greater than could be expected by chance, which is called the Euler characteristic (EC). It provides a measure of the strength of the difference between two signals (Fig 4c) and depends on the number of standard deviations chosen as a threshold. To estimate whether the EC was higher than expected by chance for a given threshold, we developed a bootstrap procedure for the GP models. We approximated a null distribution of the EC by shuffling the observed activity between the two conditions, and calculated an empirical p-value with respect to this null distribution of the EC.

If we assume a fixed threshold for calling two regions in the signal different (e.g. three s.d.), we did not find a significant difference between the two stimulus conditions (bootstrap: p ∼ 0.103, *α* = 0.01) for the calcium recordings, but for the glutamate recording (bootstrap: p ∼ 0, *α* = 0.01). Significant differences occurred during the light step and in both oscillatory sequences. The shuffle test can also be evaluated for the whole range of thresholds.

For comparison, we computed a similar test using the classical analysis pipeline, using inter-trial standard deviation as an estimate of the uncertainty associated with the mean signal. For the bootstrap procedure shuffled the interpolated data between the two stimulus conditions to approximate the null distribution. At the same threshold as above, for neither the calcium (bootstrap: p ∼ 0.062, *α* = 0.01) nor the glutamate (bootstrap: p ∼ 0.062, *α* = 0.01) recording was the EC found to be significantly elevated. It should be noted that the p-values estimated for the GP and classical pipeline are not directly comparable: the classical approach does not distinguish between observational and stimulus driven variability, rather identifying whether observed differences are greater than inter-trial variability.

The choice of a fixed threshold for inferring whether two signals are statistically distinct may result in overly conservative statistical estimates. While there were no thresholds for which the classical approach inferred an EC greater than expected from the null distribution, for the GP models of the calcium and glutamate signals there were a range of thresholds for which the EC was greater. These ranges differed between the two signals, which may relate to the effect of the physiological properties of these two signals or to the kinetics of their respective indicators on the dynamics of the observed signals. We recommend that the selection of a threshold be guided by the particular needs of the analysis task, not to keep to statistical conventions developed for other methods.

### Evaluating hierarchical clustering with GP models

We next show how GP equality tests can be used to provide a principled criterion for choosing the number of clusters in a hierarchical clustering of light responses. For example, in a single imaging plane, one may wish to know whether the observed responses originate form distinct functional groups, perhaps due to the presence of multiple cells or cell types within the recording plane, or multiple neurites of the same cell acting independently [18].

**Fig 5:**
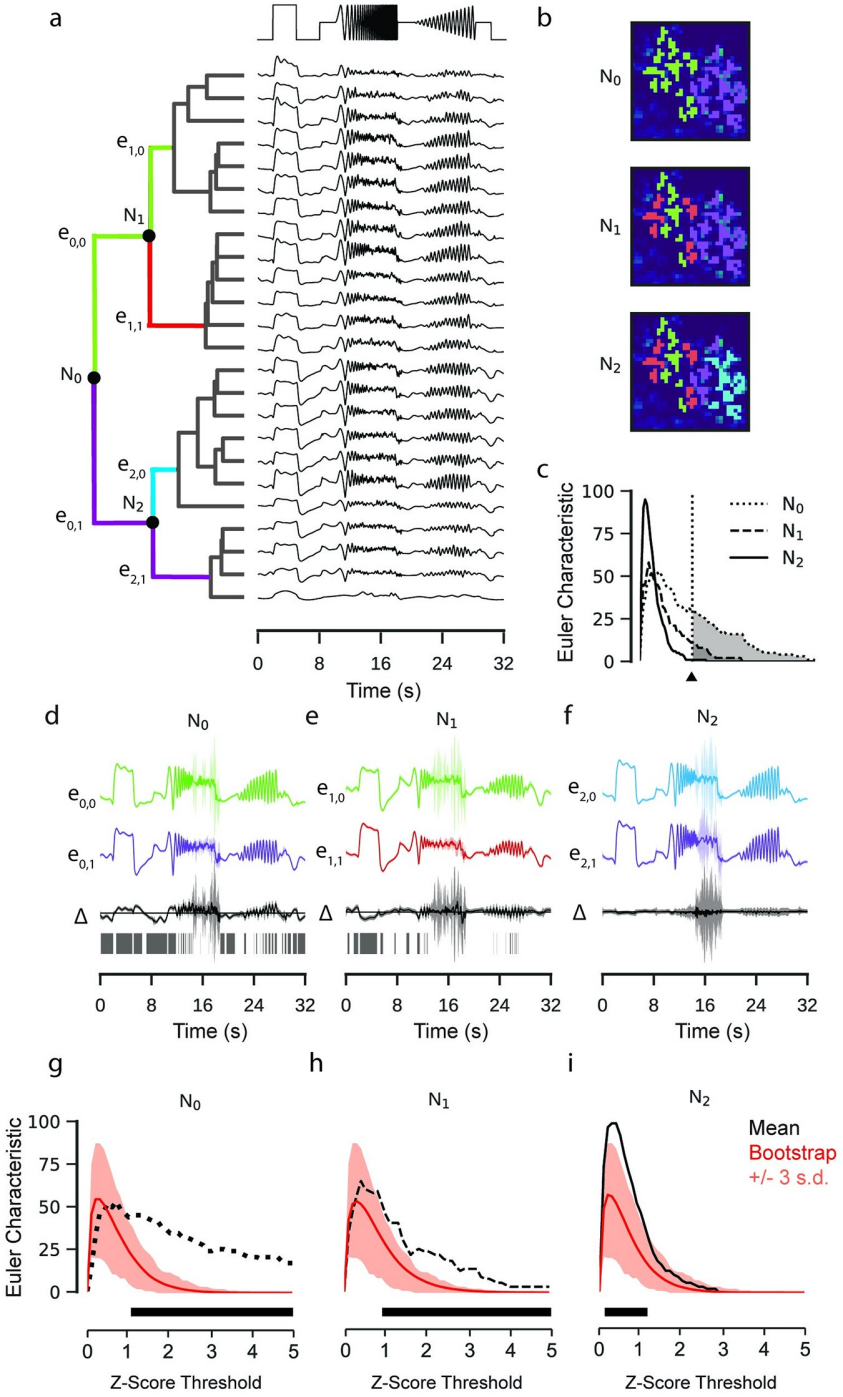
Hierarchical clustering of retinal bipolar cell terminals. Data: ROIs from a retinal bipolar cell filled with OGB-1 by sharp electrode injection recorded using a linear scan configuration. Model: RBF kernel (time), 300 inducing inputs, 20 iterations per fit, best of 3 fits per model. One model fitted for each ROI. **a**: Mean functions of GP models fitted to calcium activity in a single recording field. Dendrogram computed using the Ward’s hierarchical clustering algorithm (left). Nodes where the equality test were performed are labelled N. Colours on the dendrogram correspond to putative clusters. **b**: ROI masks overlaid on mean field activity, coloured with respect to the putative cluster. Each overlay is coloured according to the clustering at the corresponding nodes in (a). **c**: Euler Characteristic for each node with respect to the z-score threshold. **d**, **e**, **f**: GP Equality tests performed at each of the labelled nodes. GPs correspond to the models fitted to each putative cluster (top, middle) and the difference between the two models (bottom). Intervals correspond to 3 standard deviations above and below mean function. Domains where zero-vector not included within interval highlighted with grey ticks. **g**, **h**, **i**: Bootstrap estimates of the null distribution of the Euler Characteristic (EC). Mean of null distributed shown in red. Intervals correspond to three standard deviations above and below the mean of the null distribution. Estimated EC from (c) superimposed. The black box indicates the thresholds where the EC from the difference test exceeds the highest estimate from the null distribution.

This pipeline was composed of two stages, firstly identifying putative clusters, then evaluating the evidence for different cluster configurations. In the first stage, a GP was estimated for each ROI in GCaMP6f labelled bipolar cell axon terminals in a PCP2 mouse line (data previously published in [7]). Then, we hierarchically clustered the mean signals from each GP to identify putative clusters among the set of responses, using the Ward algorithm and Euclidean distance (Fig 5a).

Each node in the hierarchy then corresponded to a hypothesis about whether a particular cluster should be partitioned into two sub-clusters (Fig 5b). In the second stage, we start at the top of the clustering. At each node, we fit two GPs to the data from all ROIs assigned to each of the two clusters independently.

We then tested the hypothesis that the two clusters were different using a GP equality test with the EC as the measure of dissimilarity (Fig 5c–5f). A null distribution for the Euler Characteristic was approximated by a further bootstrap test, where the pair of signals for which the null distribution of the EC was calculated were drawn at random from the pooled observations at each node. This process continued iteratively through the hierarchy, terminating when the Euler Characteristic for a split in two new clusters was not greater than 99.5% of the null distribution at that node.

Interestingly, the first node (*N*_0_) separated ROIs belonging to two bipolar cells in the imaging field, with strong quantitative backup for the split (bootstrap: p ∼ 0 at three s.d., *α* = 0.01, Fig 5g). The split at the second node (*N*_1_) was also accepted (bootstrap: p ∼ 0, *α* = 0.01 at three s.d., Fig 5h), which separates ROIs of the left bipolar cell into two groups, indicating potential sub-clusters within the terminals of a single bipolar cell. Subsequent separations were rejected (*N*_2_, bootstrap: p ∼ 0.68, *α* = 0.01 at three s.d., Fig 5i). The difference observed within the terminal systems of these cells may reflect functional variation within the output of a single bipolar cell [19]. Were this difference to exist, it would likely be a consequence of differential inhibition from amacrine cells, and represent an additional layer of complexity in the functional parallelisation of retinal signalling. While our analysis is suggestive of this conclusion, verification is beyond the scope of this study.

### Incorporating stimulus effects into GP model inference

We next extended our GP framework to study the effect of multiple stimulus parameters and their interactions on the latent neural activity in an ANOVA-like framework [20]. GP-ANOVA models posses multiple kernels, each of which models the effect of a predictor or an interaction between predictors. In contrast to classical ANOVA, the interaction effects can have non-linear structure [20], and it is possible to compute not merely the strength of particular effects but also an inference of the response of a ROI over time as a stimulus feature varies.

**Fig 6:**
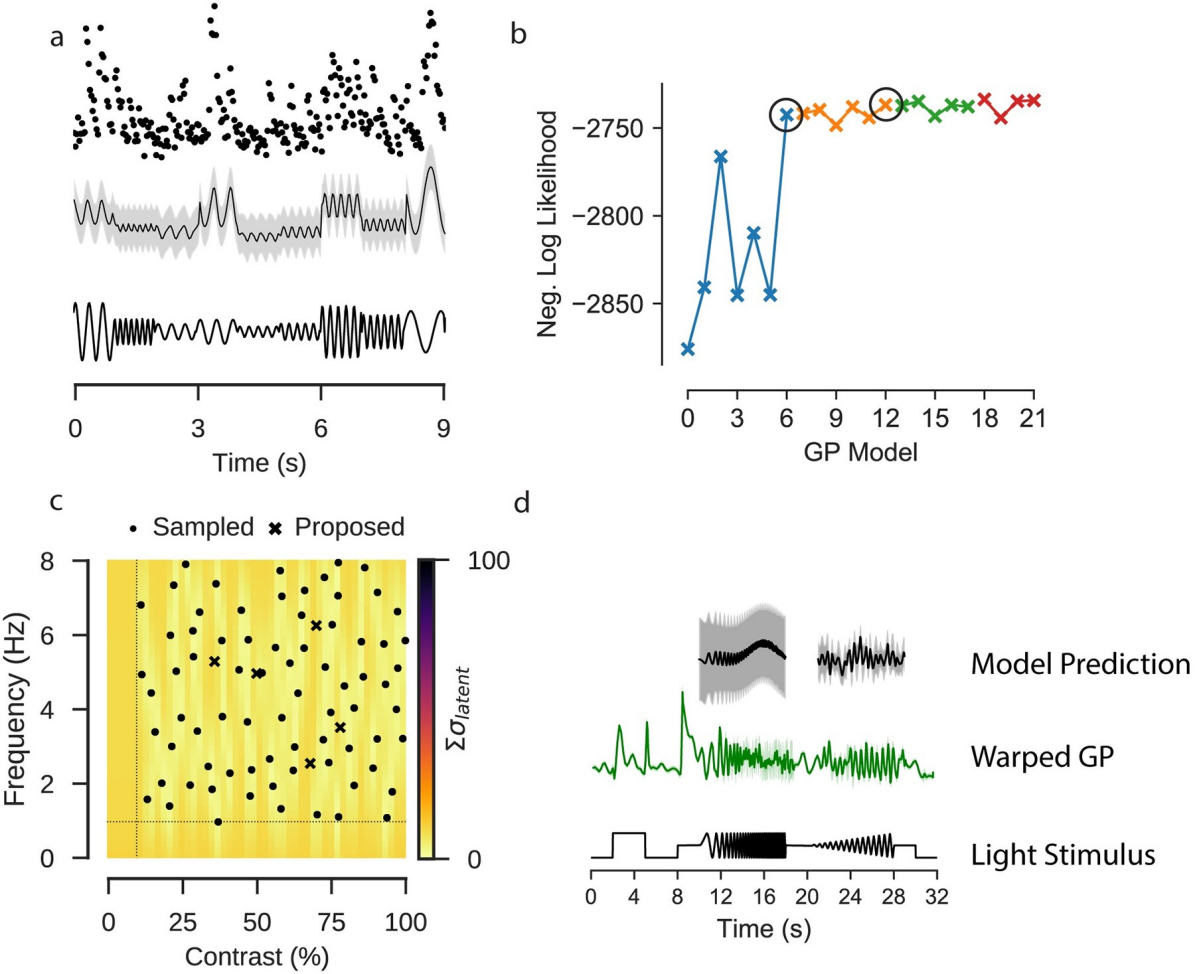
Extended GP model incorporating stimulus parameters. Data: ROI from a scan field with bipolar cell terminals in a retina expressing iGluSnFR, recorded using a spiral scan configuration. Model: Product of RBF kernel (time) with composite RBF kernels (frequency and contrast), 500 inducing inputs, 50 iterations per fit, best of 5 fits per model. **a**: Observed activity of one ROI filled with OGB-1 (top); GP model selected by model selection procedure, conditioned on the observations of the ROI. Sine stimulus activity. Data corresponds to the first 9s of the stimulus. **b**: Negative log likelihood for each model tested during model selection. Each point corresponds to a single model, where the kernel consists of the effects adopted in the previous pass, with an additional effect being evaluated, which will be included if it has the highest negative log likelihood. Each “Pass” corresponds to an exhaustive evaluation of all possible effects to add to the current kernel. The best performing model in each pass is highlighted with a black circle. **c**: Locations in frequency-contrast parameter space selected for the stimulation. Colour map corresponds to the sum of the variance of the latent function for the fitted GP model evaluated under each parameter combination. Crosses correspond to peaks in the uncertainty where the stimulus should next be evaluated. Dashed line indicates the limits of the space from which the parameters were sampled. **d**: GP fitted to observed chirp responses for the same ROI (middle). Prediction of the activity by the model on the sine stimulus data (top).

To demonstrate the usefulness of this extension, we fitted a GP model to predict the response of a single ROI to a light stimulus where light intensity was modulated as a sine wave of varying frequency and contrast (Fig 6a). The input for this model was a predictor matrix where each column corresponded to one of the stimulus parameters, including two columns jointly encoding phase as a circular feature, and one each for frequency and contrast (see Methods).

For the experiments, we selected 150 stimulus parameters using blue noise sampling, such that parameters were uniformly selected from the parameter space and excluded if they were below certain thresholds for frequency (< 1Hz) or contrast (< 10%) or too close to an already existing stimulus parameter. Although the frequency and contrast parameters are fixed during each one second trial of the sine stimulus, the model can accommodate parameters which vary continuously over time, by encoding this change in the columns of the predictor matrix.

As in a classical ANOVA, there are many possible ways in which the effects of these stimulus parameters can be incorporated into the model. In this case, stimulus features were encoded in the kernel (see Methods), either as additive independent effects of phase, contrast or frequency, or through multiplicative interactions between the features. The cost of adding more kernels with a fixed amount of data is that the uncertainty associated with each parameter increases as the number of parameters to be learned grows. To compensate for this, we performed kernel selection through a two stage iterative process (Fig 6b). The first stage identified the kernel which, when included, most strongly improved model performance, as measured by the log marginal likelihood. Once there were two or more parameters, each new kernel had to contribute a greater improvement to the model performance than could be expected by chance, as established by a likelihood ratio test (see Methods). If a kernel was accepted it was retained in the model in the consecutive iterations (for an overview of the models evaluated in this pipeline, see S1 and S2 Tables).

We fitted a GP model to the glutamate signal of a single ROI in the IPL in response to the sine stimulus using this procedure. After three iterations the improvement in model performance was less than the required ratio. We tested one further iteration which also returned a negative result, and the process ceased. The kernels which were accepted included an interaction kernel between all three parameters and a frequency-contrast kernel (Λ = 11.25, *p* < 0.001). A frequency kernel (Λ = 4.01946568, *p* = 0.045) was rejected in the third stage, and a phase kernel was rejected (Λ = 2.29, *p* = 0.130).

We then used the model to predict neural activity for unseen parameter combinations and quantified how uncertain our predictions about the activity in response to these were [21–23]. Intuitively, the model should have the least uncertainty about stimulus parameters which had been observed. Uncertainty then should increase as a function of the distance from the observed parameters. We quantified uncertainty by computing the expected response of the ROI and taking the sum of the latent variance (Fig 6c).

For the studied cell, calcium recordings to stimulation with the chirp stimulus were also available (Fig 6d), and we compared the model fitted directly to the chirp response data to predictions from the model fitted to the sine data. There were some qualitative similarities between the two models, such as the overall amplitude of the signal, and the decrease in signal amplitude as the frequency of the stimulus increased. The prediction that the signal amplitude would slightly increase with contrast was not reflected in the chirp data, where the relationship was more ambiguous. The quality of prediction of the activity at high frequencies was difficult to evaluate, as there is a high-level of uncertainty about the mean signal at those frequencies. One factor to consider with regards to this direct comparison is that the differences in the chirp responses may be due to temporal dependencies over time.

### Active Bayesian experimentation

A critical advantage of our framework is that we can use it for Bayesian experimental design. This is useful, as in 2P imaging experiments time is usually severely limited. For example, isolated mouse retinal tissue becomes unresponsive to light stimulation in a matter of hours, and single recording fields often bleach within half an hour of recording. To efficiently explore the space of possible stimulus features under severe time constraints is thus a critical problem, which GP models can be used to address [21, 22].

**Fig 7:**
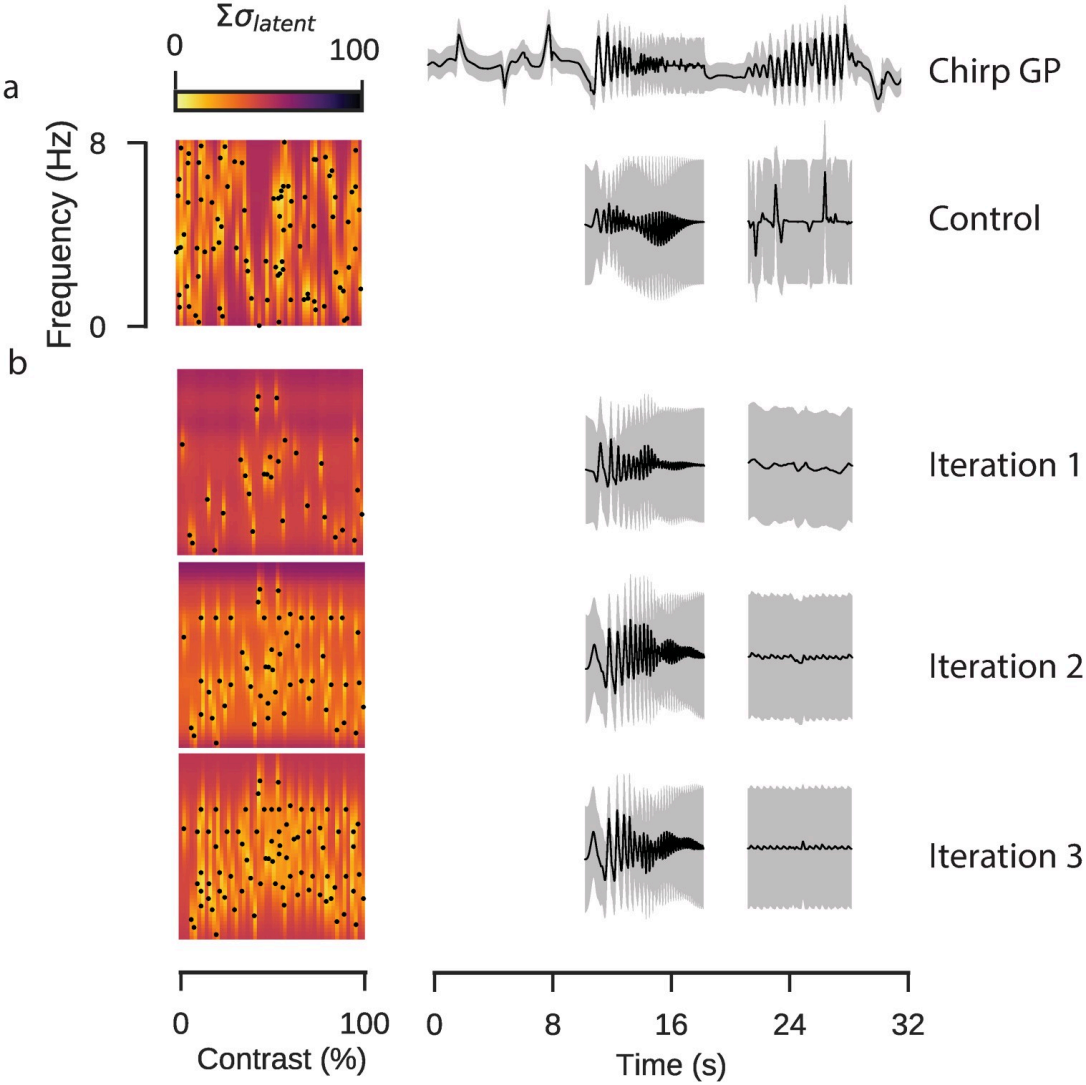
Active parameter selection with Gaussian processes. Data: ROI from a scan field with bipolar cell terminals in a retina expressing iGluSnFR, recorded using a spiral scan configuration. Model: Product of RBF kernel (time) with composite RBF kernels (frequency and contrast), 500 inducing inputs, 50 iterations per fit, best of 4 fits per model. **a**: Control stimulus consisting of 90 parameter sets of frequency and contrast. Uncertainty in each region computed as the sum of the latent uncertainty for a GP estimated under all parameter configurations. The chirp response for this ROI is shown above. The completed GP model for the sine response is estimated over the full dataset, the model inference for the sinusoidal chirp components is shown. **b**: Active parameter selection using GP with corresponding inferences for chirp stimulus.

To show how this works, we performed an experiment using GP models to guide parameter selection. In retinal tissue expressing iGluSnFR we selected a single ROI, likely representing a single bipolar cell axon terminal. We used two control stimuli to evaluate the parameter selection: a local chirp stimulus playing over three trials, to which we fitted a warped GP, and a sinusoidal stimulus with 90 parameters uniformly sampled from the parameter space (Fig 7a), to which we fitted a GP with the kernels derived in the previous likelihood ratio procedure. We performed three rounds of active parameter selection, starting with 30 uniformly sampled parameters in the first iteration, fitting the GP and using parameters selected by identifying 30 peaks in the uncertainty map in the subsequent two iterations. We then used the models from each iteration to predict how the ROI would respond during the oscillatory components of the chirp stimulus (Fig 7b).

Parameters selected using the active approach were more broadly distributed across the parameter space, although we noted that the peak finding algorithm was biased away from the edges. In the purely random design procedure, parameters often clustered and there were large empty regions, resulting in high uncertainty in these regions. Neither the random nor the active parameter procedure inferred a good prediction of the contrast-varying chirp component, which in the case of the active parameter inference was likely due to the bias away from the periphery of the parameter space, resulting in very few samples in the proximity of the 8Hz parametric edge. At lower frequencies the experimental design algorithm seemed better able to capture qualitative aspects of the chirp response, such as the decrease in response amplitude as the frequency increased, though again the lack of samples at the very highest frequencies resulted in a high level of uncertainty.

### Combining model components

**Fig 8:**
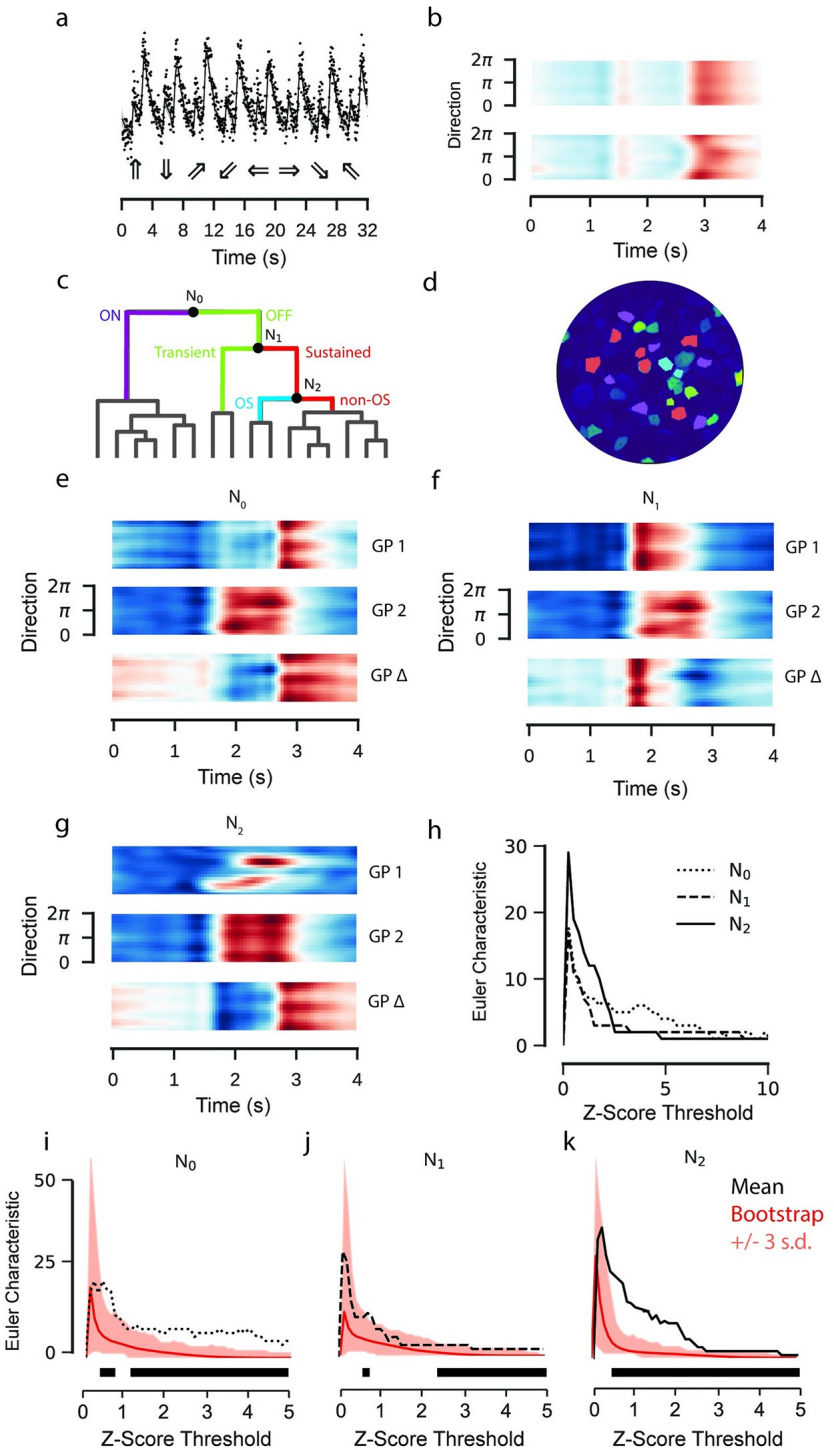
GP model of retinal ganglion cell responses to a moving bar stimulus. Data: ROIs from a field of RGC somata labelled with OGB-1 using electroporation, recorded using a spiral scan configuration. Model: Product of RBF kernel (time) with composite RBF kernel (direction), 300 inducing inputs, 25 iterations per fit, best of 3 fits per model. **a**: Observed activity of one ROI representing an RGC soma labelled with OGB-1 (top). GP model superimposed. Intervals correspond to the variance of the latent function, 3 standard deviations above and below the mean. Below are the moving bar directions for each trial. **b**: GP model fitted to data in (a) without (top) and with (bottom) interaction kernel. Both models include additive effects for direction and time. Coloured according to response amplitude (red: high, blue: low). **c**: Hierarchical clustering of fitted models. Colours on dendrogram correspond to colours on ROI mask. Posterior means for each ROI in each cluster are shown in S2 Fig. **d**: ROI mask with cluster colours for *k* = 4 corresponding to clustering in (c). A baseline s.d. was computed from the 500 ms of activity preceding the light step. ROIs where the amplitude of the step response was less than 2 s.d. greater or lower than the mean signal were excluded. **e**, **f**, **g**: GP Equality Tests for each node. Top and middle correspond to the two putative clusters, bottom is the mean difference between them. **h**: Euler Characteristic as a function of the number of standard deviations above and below the respective mean functions, for each node. **i**, **j**, **k**: Euler Characteristics (EC) from difference tests at the first three nodes, with bootstrap estimates of the null distribution of the EC superimposed. The mean of the null distributed shown in red. Intervals correspond to three standard deviations above and below the mean of the null distribution. The black bar indicates the thresholds where the EC from the difference test exceeds the highest estimate from the null distribution by three standard deviations.

We finally constructed a model which combined stimulus effect modelling and hierarchical clustering into a single framework. We fitted the model to calcium recordings of RGC activity in response to a bright bar moving in different directions on a dark background. RGCs show different response polarities and a large range of response kinetics to this stimulus [11] and some modulate the response amplitude as a function of stimulus direction. The model incorporated the stimulus features of time and direction as additive effects, alongside with a time-direction interaction effect (Fig 8a and 8b). The data were then sorted using hierarchical clustering (Fig 8c and 8d; S2 Fig) and for the purpose of demonstration the first three nodes of the hierarchy were tested using GP equality tests (Fig 8e–8h).

The algorithm first separated ON and OFF responses into separate clusters (*N*_0_, bootstrap: p ∼ 0 at three s.d., Fig 8i). The ON cluster was then further divided into sustained and transient responses (*N*_1_, bootstrap: p ∼ 0 at three s.d., Fig 8j). The sustained ON responses were finally separated into direction selective and non-direction selective clusters (*N*_2_, bootstrap: p ∼ 0.01 at three s.d., Fig 8k). We did not test further splits for significance.

## Discussion

Here we presented a data analysis pipeline for 2P imaging data based on Gaussian Processes. The advantage of this framework is that uncertainty about the underlying latent neural activity can be propagated through the analysis pipeline, so statistical inference can be performed in a principled way. We applied our pipeline to recordings of mouse retinal bipolar and ganglion cell activity driven by light stimuli, showing how: (1) to determine whether and when two response functions are statistically distinct; (2) to evaluate the strength of the evidence for a partition of data into functional clusters; (3) to determine the relevant stimulus effects to incorporate into a model of neural responses; and (4) to guide the choice of stimulus parameters for iterations of a closed loop adaptive experiment.

### Estimation of uncertainty

Accurately characterising the variability of neural responses is essential for understanding neural coding. Noise manifests itself throughout sensory systems and presents a fundamental problem for information processing [2]. While imaging *ex-vivo* retinal tissue does not present some of the challenges as *in vivo* cortical recordings (where movement is a significant source of variability), two-photon imaging in *ex-vivo* tissue is still subject to many sources of variance, due to fluctuations in biosensor excitation and photon detection, among other factors. This issue may be particularly acute for two-photon imaging of retinal tissue, where it is necessary to keep the excitation energy low to avoid laser-evoked responses, which may result in lower overall fluorescent signals relative to in vivo recordings of non-light-sensitive tissue. Computational processing can introduce further variability, e.g. due to the discretisation of the measured signal. This is rarely acknowledged, perhaps due to the convenience of standard approaches. In principle, splines in combination with generalized additive models (GAMs, e.g. [24]) provide an alternative framework to perform uncertainty aware analysis of calcium imaging data. Exploring and contrasting this to the GP framework introduced here is beyond the scope of this paper.

### Computational limitations of the proposed models

Classical GP models can be computationally costly due to the need to compute the inverse of the kernel matrix involving all training data [4]. To make practical use of GPs for modelling large 2P recordings, we capitalized on recent advances in sparse approximations for GPs that work with a limited number of inducing points [5] and demonstrated their applicability for a real world task. In addition, we only performed point-estimates for hyperparameters instead of fully Bayesian inference and pre-determined kernels before statistical evaluation. This was important for two reasons: firstly, a processing pipeline should not be excessively computationally costly, so as to make them impractical for general use with larger datasets; and secondly, the application of these models in closed-loop imaging experiments was only possible if one complete iteration of the process (data acquisition; pre-processing; prediction; parameter selection) could be completed in a few minutes. In principle, our approach could be extended to a fully Bayesian framework with hyperpriors on the model parameters, although this introduces additional difficulties for sparse approximation and still entails a greater computational burden [25]. While our work solely addressed Gaussian distributed data, the models can be readily extended to point processes as well. There, sparse approximation techniques overcome the computational intractability of the model, and allow inference on relatively large datasets [26].

### Active experimental design of 2P experiments

Although we demonstrated the potential for using GP models during 2P experiments, there were several limitations to our approach. We were able to reduce the time per iteration of our active experiments to less than five minutes, addressing a key practical concern. However, it emerged during the experiment that the peak-finding algorithm was biased away from the periphery of the stimulus space, which made the chirp stimulus unsuitable as our “ground truth” for model evaluation.

The parameter batch size may also have been too small for each iteration. Batch size is a critical consideration in active Bayesian experimentation. Where the cost per iteration is low, single parameters can be selected for each iteration, for which the objective function can be relatively easily defined and evaluated. In one recent publication, Charles et al. [23] used GPs to model the effect of inter-trial variability in monkey V1 neurons, using sequential parameter selection to optimise a coloured light stimulus. For experiments where iterations are prohibitively expensive, new parameters can be selected in batches, although this requires interactions between parameters to be taken into account, which can be computationally expensive to evaluate. In such cases, approximate methods provide an attractive method for reducing computational overheads (e.g. [22]). Batch parameter selection algorithms which account for, or approximate, parameter interactions would likely overcome simple peak finding methods.

### Conclusion

Historical obstacles to the use of Bayesian methods such as the difficulty of their implementation and their computational cost have been reduced. Much research over the past decade has focused on the problem of minimising the computational complexity of the algorithms through sparse approximation methods and efficient parameter estimation (such as [5]). New libraries for popular coding languages—such as GPy [27], PyMC3 [28], and pySTAN [29] for Python 3—have lowered the barrier to entry. Likewise, we provide a collection of notebooks with this paper to allow straightforward application of our framework.

Taken together, our approach exploits the flexibility and extensibility of Gaussian process models to improve on classical approaches for two photon data analysis and addresses important analytical tasks in a way that preserves a representation of uncertainty propagated up from the underlying data. We feel that it will be particularly useful for disentangling the dynamics of neural circuits in the early visual system under complex, multivariate experimental conditions.

## Materials and methods

### Ethics statement

All animal procedures were performed according to the laws governing animal experimentation issued by the German Government. The documentation for the animal and tissue preparation was submitted in accordance with Mitteilung nach §4 Abs. 3 Tierschutzgesetz, and approved by the Regierungspräsidium Tübingen on 09.11.2016. Viral injection documentation Tierversuch Nr. AK6/13 was appraised by the ethics committee and approved by Regierungspräsidium Tübingen, on 05.11.2013.

### Animals and tissue preparation

For single-cell-injection experiments, we used one adult mouse cross-bred between transgenic line B6.Cg-Tg(Pcp2-cre)3555Jdhu/J (Tg3555, JAX 010536) and the Cre-dependent red fluorescence reporter line B6;129S6-Gt(ROSA)26Sor^tm9(CAG-tdTomato)Hze^/J (Ai9^tdTomato^, JAX 007905). For glutamate-imaging, we used one adult C57BL/6J mouse. Owing to the exploratory nature of our study, we did not use blinding and did not perform a power analysis to predetermine sample size.

Animals were housed under a standard 12h day-night cycle. For recordings, animals were dark-adapted for ≤ 1h, then anaesthetised with isoflurane (Baxter) and killed by cervical dislocation. The eyes were removed and hemisected in carboxygenated (95% O_2_, 5% CO_2_) artificial cerebral spinal fluid (ACSF) solution containing (in mM): 125 NaCl, 2.5 KCl, 2 CaCl_2_, 1 MgCl_2_, 1.25 NaH_2_PO_4_, 26 NaHCO_3_, 20 glucose, and 0.5 L-glutamine (pH 7.4). Then, the tissue was moved to the recording chamber of the microscope, where it was continuously perfused with carboxygenated ACSF at ∼ 37°C. The ACSF contained ∼ 0.1*μ*M sulforhodamine-101 (SR101, Invitrogen) to reveal blood vessels and any damaged cells in the red fluorescence channel. All procedures were carried out under very dim red (>650nm) light.

### Single cell injection

Sharp electrodes were pulled on a P-1000 micropipette puller (Sutter Instruments) with resistances between 70–100MΩ. Afterwards, the tip (∼ 500*μ*m) of each electrode was bent on a custom-made microforge. Single bipolar cell somata in the inner nuclear layer were filled with the fluorescent calcium indicator Oregon-Green BAPTA-1 (OGB-1) by using the pulse function (500ms) of the MultiClamp 700B software (Molecular Devices). OGB-1 (hexapotassium salt; Life Technologies) was prepared as 15mM in distilled water. Immediately after filling, the electrode was carefully retracted. Imaging started after about 30 minutes after the injection to allow cells to recover and the dye to diffuse within the cell. At the end of the recording, a stack of images was captured for the cellular morphology, which was then traced semi-automatically using the Simple Neurite Tracer plugin implemented in Fiji [30].

### Virus injection

For virus injections, we used adult wild-type mice (C57BL/6J). Animals were anesthetized with 10% ketamine (Bela-Pharm GmbH & Co. KG) and 2% xylazine (Rompun, Bayer Vital GmbH) in 0.9% NaCl (Fresenius). A volume of 1*μ*l of the viral construct (AAV2.hSyn.iGluSnFR.WPRE.SV40, Penn Vector Core) was injected into the vitreous humour of both eyes via a Hamilton injection system (syringe: 7634-01, needles: 207434, point style 3, length 51mm, Hamilton Messtechnik GmbH) mounted on a micromanipulator (World Precision Instruments). Imaging experiments were performed 3 weeks after virus injection.

### Two-photon imaging

We used a MOM-type 2P microscope (designed by W. Denk, now MPI Martinsried; purchased from Sutter Instruments/Science Products). The design and procedures have been described previously [7, 11, 31]). In brief, the system was equipped with a mode-locked Ti:Sapphire laser (MaiTai-HP DeepSee, Newport Spectra-Physics), two fluorescence detection channels for OGB-1 or iGluSnFR (HQ 510/84, AHF/Chroma) and SR101/tdTomato (HQ 630/60, AHF), and a water immersion objective (W Plan-Apochromat 20x /1.0 DIC M27, Zeiss). The laser was tuned to 927nm for imaging OGB-1, iGluSnFR or SR101. For image acquisition, we used custom-made software (ScanM by M. Müller, MPI Martinsried, and T. Euler) running under IGOR Pro 6.3 for Windows (Wavemetrics), taking time lapsed 32 x 32 pixel image scans (at 15.625Hz) or 16-line “spiral” scans (at 31.25Hz). For documenting morphology, 512 x 512 pixel images were acquired with step size of 0.5*μ*m along the Z axis.

### Fast spiral scan imaging

To resolve transient changes in calcium concentration or glutamate release (i.e. with decay times of ∼100ms), scan rates of around 20Hz or more are wanted. Many scanning 2P microscopes use conventional (non-resonant) galvanometric scanners and are limited by the inertia of the scan mirrors, which introduce positional errors at high scan rates. This is especially critical for typical linear (image) scans, with their abrupt changes in direction when jumping between scan lines. For constant spatial resolution, faster scan rates are often realised by decreasing the scan area. However, it is possible to increase the spatio-temporal resolution by using non-linear “spiral scan” configurations. These overcome the key mechanical limitation of linear scans, that they incorporate sharp turns, rather than following smoother trajectories. Unlike linear scans, which are composed of single linear trajectories repeated along an axis at regular intervals, spiral scan configurations consist of radial trajectories moving away from a central point at a constant speed and rotation and permit rapid movement of the scan mirrors.

A regular radial grid can be constructed by generating a single spiral trajectory and successively rotating it around a central point. We used an Archimedean spiral is used to generate each trajectory (*r* = Θ^1/*a*^), where the radial distance *r* from the central point is a function of the angle Θ and a tightness parameter *a* which determines the rate of rotation around the centre. With a grid composed of 16 such curves we can resolve, for instance, axon terminals of retinal bipolar cells at twice the spatial and twice the temporal resolution of linear recordings. One can see the advantages of such scan configurations by showing how frequently the scan trajectory intersects with ROIs in a single frame. The times at which labelled structures are observed by these trajectories are both more frequent and more irregularly distributed in time than a typical linear scan, providing a superior temporal resolution.

### Light stimulation

For light stimulation, a modified LightCrafter (DLPLCR4500, Texas instruments; modification by EKB Technology) was focused through the objective lens of the microscope. Instead of standard RGB light-emitting diodes (LEDs), it was fitted with a green (576nm) and a UV (390nm) LED for matching the spectral sensitivity of mouse M- and S-opsins [32]. To prevent the LEDs from interfering with the fluorescence detection, the light from the projector was band-pass-filtered (ET Dualband Exciter, 380-407/562-589, AHF) and the LEDs were synchronised with the microscope’s scan retrace. Stimulator intensity was calibrated to range from 0.5 * 10^3^ (“black” background image) to 20 * 10^3^ (“white” full field) photoisomerisations P*/s/cone [7]. The light stimulus was centred before every experiment, such that its centre corresponded to the centre of the recording field. In linear scans, the stimulus is displayed while the trajectory moves between consecutive lines; while for the spiral scans this occurs while the trajectory returns from the periphery to the centre.

Light stimuli were generated using the QDSpy light stimulation software, which is written in Python 3 [33]. The chirp stimulus ran for 4 repeats of 32s each, with the stimulus extent alternating between a 800*μm* and a 100*μm* light spot. The moving bar stimulus consisted of a 300*μm* rectangular bar moving at 1000*μm*/*s* for 4 seconds along 8 evenly space directions, repeated three times for each direction. The sine stimulus consisted of a 100*μm* light spot, and ran for 45 1s-trials, with contrast and frequency varying in each trial. The contrast and frequency parameters were chosen by blue-noise sampling 150 parameters from the parameter space, between 10% and 100% contrast and 1Hz to 8Hz frequency. Later closed-loop experiments used a sine stimulus with 90 parameter sets sampled uniformly from the parameter space, in addition to 3x30 parameters sets, of which the first were chosen from random uniform sampling and the latter two sets by active Bayesian inference.

### Data analysis

Initial data analysis was performed in IGOR Pro 6. Regions of Interest (ROIs) were defined manually using the SARFIA toolbox for IGOR Pro [34]. In the iGluSnFR recordings, a custom-script generated a correlation map [7], which defined structures for the ROI drawing. The observations were synchronised to the light stimuli using time markers which were generated by the stimulation software and acquired during imaging. Once the initial pre-processing was completed, the data was exported to HDF5 files, and all subsequent analysis was performed in Python 3.5.

### Gaussian process models

Gaussian process (GP) models were used to infer the relationship between time, stimulus parameters and the observed activity of each ROI. Thus, the predictor matrix *X* was a function of the stimulus parameters and time, short hand referred to as *θ*. An introduction to the mathematics of GP regression is provided in Box 2. All GPs used the Radial Basis Function (RBF) kernel, with additive Gaussian noise.

$$k_{RBF,\phi }\left(X,X^{\prime }\right)=\sigma_{signal}^{2}\text{exp}\left(-\frac{∥X-X^{\prime }{∥}^{2}}{2l^{2}}\right)+I\sigma_{noise}^{2}$$ (10)

$$\phi =\{l,\sigma_{signal},\sigma_{noise}\}$$ (11)

The lengthscale *l*, signal variance *σ*_*signal*_ and noise variance *σ*_*noise*_ were learned as part of the model optimisation. Since the fluorescence measurements *F*_*i*,*θ*_ for ROI *i* were irregularly spaced in time, the mean *μ* and covariance Σ of the signal *F*_*i*_ were inferred for a new set of predictors X* where time is regularly spaced:

$$\mu_{\phi }\left(X^{*}|X\right)=k_{\phi }\left(X^{*},X\right){\left(k_{\phi }\left(X,X\right)+I\sigma_{noise}^{2}\right)}^{-1}F_{i,\theta }$$ (12)

$$\begin{array}{c} \Sigma_{\phi }\left(X^{*}|X\right)=k_{\phi }\left(X^{*},X^{*}\right)-\\ k_{\phi }\left(X^{*},X\right){\left(k_{\phi }\left(X,X\right)+I\sigma_{noise}^{2}\right)}^{-1}k_{\phi }\left(X,X^{*}\right) \end{array}$$ (13)

The additive noise component $I\sigma_{noise}^{2}$ was removed for statistical inference, and we refer to the resultant noise-free GP as the “latent function”, in line with the terminology in the GPy documentation [27]. Confidence intervals were calculated as

$$\mu_{\phi }\left(X^{*}\right)\pm 3*diag\left(\Sigma_{\phi }\left(X^{*}\right)\right)$$ (14)

The Gaussian process models were developed in the GPy framework [27]. Feature encoding, input warping, equality tests, parameter selection and closed-loop parameter selection were computed using custom scripts, which we provide as supplementary content to this document and online at https://github.com/berenslab/bayesian_2p_pipeline. Hierarchical clustering was performed using scripts from the Scipy library, using Euclidean distance, the Ward algorithm and maxclust as the criteria [35]. The Ward algorithm was chosen as it tends to infer balanced clusters across the hierarchy. Adaptive parameter selection used a local peak finding algorithm from the Scikit-Image library.

Since our datasets included several thousand observations, it was necessary to use sparse approximation methods to fit the GP models. The sparse approximation algorithm provided in GPy follows [5], whereby the kernel is approximated using a subset of the data, termed the inducing inputs. The selection of the inducing inputs is learned as part of the model optimisation, selecting the inputs which minimise the KL-Divergence between the approximation and the target distribution. Details are provided in [5].

### Gaussian process equality tests

The Gaussian process equality test establishes whether two functions modelled by GPs are equal [14]. It operates by computing the difference between the two distributions and identifying whether the credible region encompasses the zero vector across the complete domain of the predictors. If the zero vector is outside of these intervals, we say the two functions are distinct with probability 1 − *a*. The probability is calculated using the mean μ* and covariance k* of the posterior of our two functions, excluding their respective noise components from the estimate of the covariance.

$$\mu_{\phi }^{\Delta }(X^{*}|X)\mu_{\phi }{}^{T}\Sigma_{\phi }^{\Delta }(X^{*}|X)\Sigma_{\phi }\mu_{\phi }^{\Delta }(X^{*}|X)\leq \chi^{2}\left(1-a\right)$$ (15)

$$\mu_{\phi }^{\Delta }\left(X^{*}|X\right)=\mu_{\phi }^{1}\left(X^{*}|X\right)-\mu_{\phi }^{2}\left(X^{*}|X\right)$$ (16)

$$\Sigma_{\phi }^{\Delta }\left(X^{*}|X\right)=\Sigma_{\phi }^{1}\left(X^{*}|X\right)+\Sigma_{\phi }^{2}\left(X^{*}|X\right)$$ (17)

### Euler characteristic bootstrap

The total number of discrete, non-intersecting regions where two Gaussian processes differ more than could be expected by chance is termed the Euler characteristic (EC) [36]. The EC is a measure of the geometry of random fields which accounts for the smoothness of the underlying functions, and is well established in fMRI research, where it forms part of the broader literature on statistical mapping [37]. While the expected value of the EC can be analytically tractable under certain conditions, we wished to incorporate it into our pipeline in a manner which was not sensitive to the number of input dimensions and could handle non-stationary autocorrelation functions, and so inferred its null distribution through bootstrap resampling.

To evaluate whether the values of the EC, which were obtained from the GP equality tests, were statistically significant, we constructed an approximate null distribution by bootstrapping samples from pooled data and performing equality tests on these samples. The procedure was as follows: the observations from each of the signals being compared were pooled to form a larger set of observations; from this set, pairs of samples each 300 observations in size were drawn at random, without replacement; Gaussian processes were fitted to each of the samples in the pair; the difference between the two Gaussian process models was calculated; the Euler characteristic was calculated from this difference for varying thresholds. This was repeated 500 times to build an approximate null distribution.

We applied this bootstrap test in Figs 4, 5 and 8. For Fig 4, the observations were pooled from the responses to the stimulus; for Figs 5 and 8, for each node the observations were pooled from the two putative clusters. For the comparisons to the classical pipeline, the null distribution was computed by shuffling the observations between stimulus conditions, with a total of 500 shuffled pairs used for the estimation. Approximate p-values were computed by calculating the proportion of the *N* shuffled sample pairs which had a greater EC value than that calculated from the GP equality test.

### Non-stationary autocorrelation

To address non-stationarity of the chirp response data, we computed the autocorrelation function for the chirp stimulus in 500 ms windows spaced at 1/16 s intervals (512 windows total). As we used RBF kernels for our regression, we fitted a Gaussian curve to each autocorrelation function and retained the inferred lengthscale *l*_*t*_ for each window. A further parameter *A* modulates the height of the function.

$$cov\left(f_{stimulus}\right)∼Ae^{\frac{{\left(x-\mu \right)}^{2}}{2l_{t}^{2}}}$$ (18)

If the signal were stationary, we would observe that the lengthscale parameter was constant with respect to time.

By using the cumulative sum of the inverse of the lengthscale as the predictor, we could derive a warping function which transformed the predictors such that the stimulus autocorrelation was stationary.

We assumed that the autocorrelation of the observed signal was approximately equal to that of the light stimulus input, and used the warping function to transform the observations. This transformation could be inverted to visualise the fitted GP with respect to the original time base.

$$f_{warped}\left(x_{t}\right)∼\frac{1}{2l_{t}^{2}}$$ (19)

### Gaussian process ANOVA

GP models can also be used for functional Analysis of Variance [20]. These GP-ANOVA models disentangle the contribution and interaction of different predictors to the observed function. The GP models for the chirp stimulus data modelled the observed activity with time relative to the start of each stimulus trial as the predictor *X*. For the moving bar stimulus, a direction parameter was encoded as a 2D circular feature by converting the angle *α* in polar coordinates to an xy position in Cartesian coordinates (*cos*(2*πα*/360), *sin*(2*πα*/360)). Likewise, for the sine wave stimuli the phase of the oscillation was encoded as (*cos*(2*πtf*), *sin*(2*πtf*)), while frequency and contrast were encoded linearly.

In contrast to classical ANOVA, the effects and interactions can be non-linear. Flexible kernel composition makes such models comparatively simple to implement. Kernels can be combined in a number of ways [4], each expressing some belief about the effect of a parameter, most commonly by taking the sum or product of two kernels. Additive components represent effects of predictors which are independent of one another, while multiplicative kernels represent interactions between predictors. For example, for a kernel encoding two stimulus parameters *x*_*a*_ and *x*_*b*_, with both additive and interactive effects and RBF kernels, the correlation function of the GP model would be:

$$k_{\phi }\left(X,X^{\prime }\right)=k_{\phi }\left(x_{a},x_{a}^{\prime }\right)+k_{\phi }\left(x_{b},x_{b}^{\prime }\right)+k_{\phi }\left(X_{a,b},X_{a,b}^{\prime }\right)$$ (20)

Here, *X*_*a*,*b*_ = (*x*_*a*_, *x*_*b*_). We estimated interaction effects of different stimulus parameters in our GP ANOVA models by including kernels which learned a single lengthscale parameter over multiple input dimensions. This inferred the joint effect of the parameters as a single function; where, since the parameters are z-scored, a change in the magnitude of one stimulus parameter would have the same effect as varying the other by an equal magnitude. This approach to GP ANOVA provides an efficient and principled way of choosing optimal hyperparameters to infer stimulus effects.

For the chirp stimulus, where there is one predictor, a single kernel encoding the autocorrelation of the signal over time was used. For the warped GPs, the warped time was used instead. For the moving bar and sine wave stimuli, additional kernels were included to model the effects of their respective parameters. The GP model for the moving bar responses included both additive effects for time and direction, and a time-direction interaction effect. Likelihood ratio tests were used to select kernels from the full set of additive and multiplicative stimulus effects:

$$\chi^{2}=-2ln\left(\frac{L_{0}}{L_{1}}\right)$$ (21)

Where *L*_*N*_ is the likelihood of the fitted model, and the addition of the proposed parameter is rejected if the improvement in the likelihood is greater than chance with probability 1 − *a*. These tests were applied iteratively until a kernel was rejected. For the closed loop experimentation, we retained the model from the previous selection procedure with the randomly parameterised sine stimulus.

The data used throughout this paper and corresponding code used to compute the models will be provided as supplementary material alongside this paper.

## Supporting information

S1 Fig Model performance. Model: RBF kernel, best of 5 fits per model. **a**: Mean time elapsed per iteration of the MLE. This scales approximately linearly with the number of points. **b**: Out of sample estimate of negative log likelihood after 30 iterations. Estimates as a function of the number of inducing points. **c**: Model performance of out of sample test points relative to the maximum number of iterations, evaluated for 300 inducing points.
(PNG)

S2 Fig Direction selectivity clusters. Model: Product of RBF kernel (time) with RBF kernel (direction), 300 inducing inputs, 25 iterations per fit, best of 3 fits per model. Each heat map corresponds to the posterior mean of the fitted GP. Columns correspond to clusters.
(PNG)

S1 Table Oscillatory kernels. Each kernel incorporates either independent or interactive effects of the stimulus parameter into the prediction. Kernels were selected by a sequence of likelihood ratio tests, the results of which are shown in S2 Table.
(ODS)

S2 Table Oscillatory models. Results from the sequential likelihood ratio test from which the stimulus parameter model was constructed for the sinusoidal stimulus data.
(ODS)
