# Correction: Bayesian hypothesis testing and experimental design for two-photon imaging data

**DOI:** 10.1371/journal.pcbi.1007473

**Published:** 2019-10-22

**Authors:** Luke E. Rogerson, Zhijian Zhao, Katrin Franke, Thomas Euler, Philipp Berens

There is an error in the Funding statement. One of the grant numbers is incorrect and should be BE 5601/4-1 instead of BE 5604/1-1.

The corrected statement should read: “This research was supported by German Research Foundation (DFG; BE 5601/4-1 to PB; Cluster of Excellence “Machine Learning—New Perspectives for Science, EXC 2064 to PB, project number 390727645; SFB 1233 "Robust Vision" to PB and TE—project number 276693517), the Federal Ministry of Education and Research (BMBF; FKZ 01GQ1002 to TE and KF, FKZ 01GQ1601/Bernstein Award to PB), the National Eye Institute (NEI; 1R01EY023766-01A1 to TE) and the Max Planck Society (MPG; M.FE.A.KYBE0004 to KF). The funders had no role in study design, data collection and analysis, decision to publish or preparations of the manuscript.”
