# Supplementary figures and images for: Bayesian hypothesis testing and experimental design for two-photon imaging data
Source: PLoS Comput Biol. 2019 Aug 2;15(8):e1007205. doi: 10.1371/journal.pcbi.1007205 (PMC6693774; doi:10.1371/journal.pcbi.1007205)

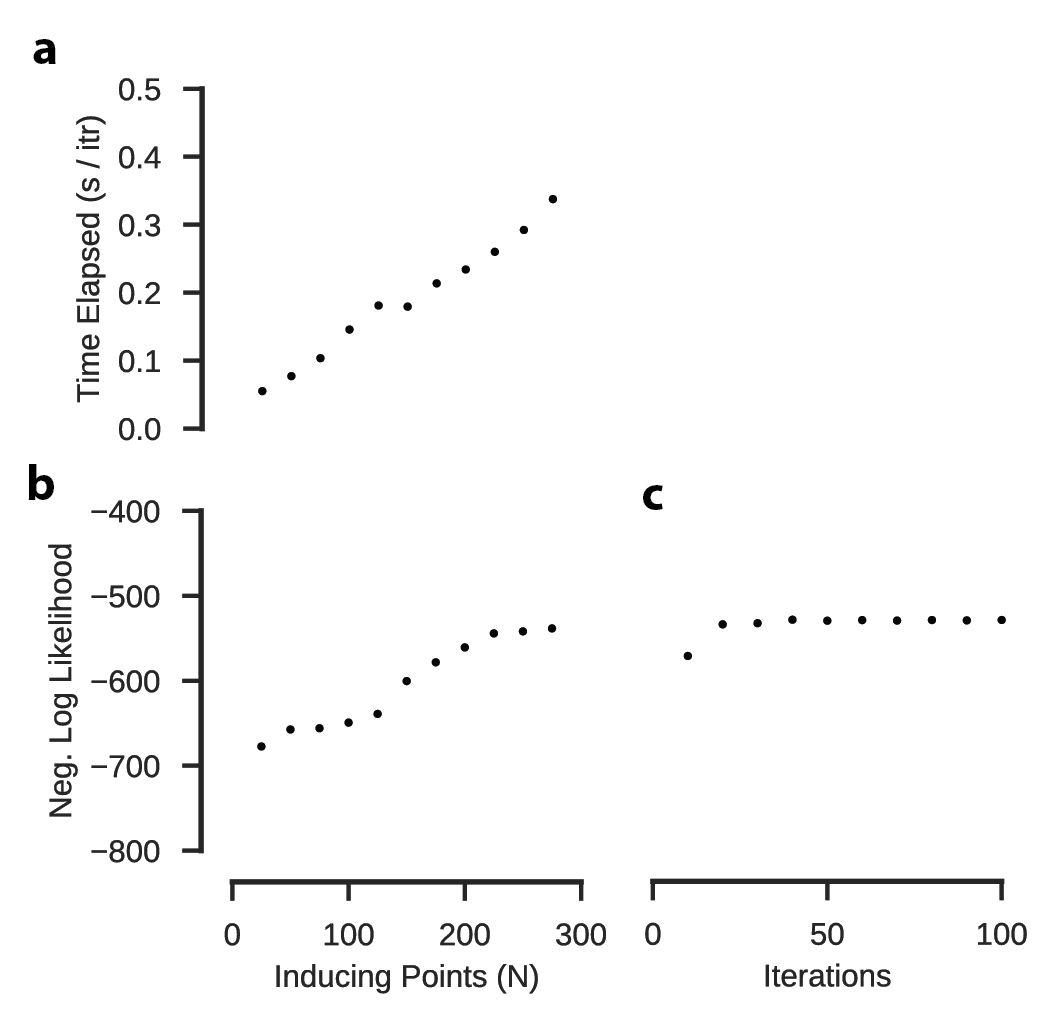

Supplement: S1 Fig — Model: RBF kernel, best of 5 fits per model. a: Mean time elapsed per iteration of the MLE. This scales approximately linearly with the number of points. b: Out of sample estimate of negative log likelihood after 30 iterations. Estimates as a function of the number of inducing points. c: Model performance of out of sample test points relative to the maximum number of iterations, evaluated for 300 inducing points. (PNG) [file pcbi.1007205.s001.png]

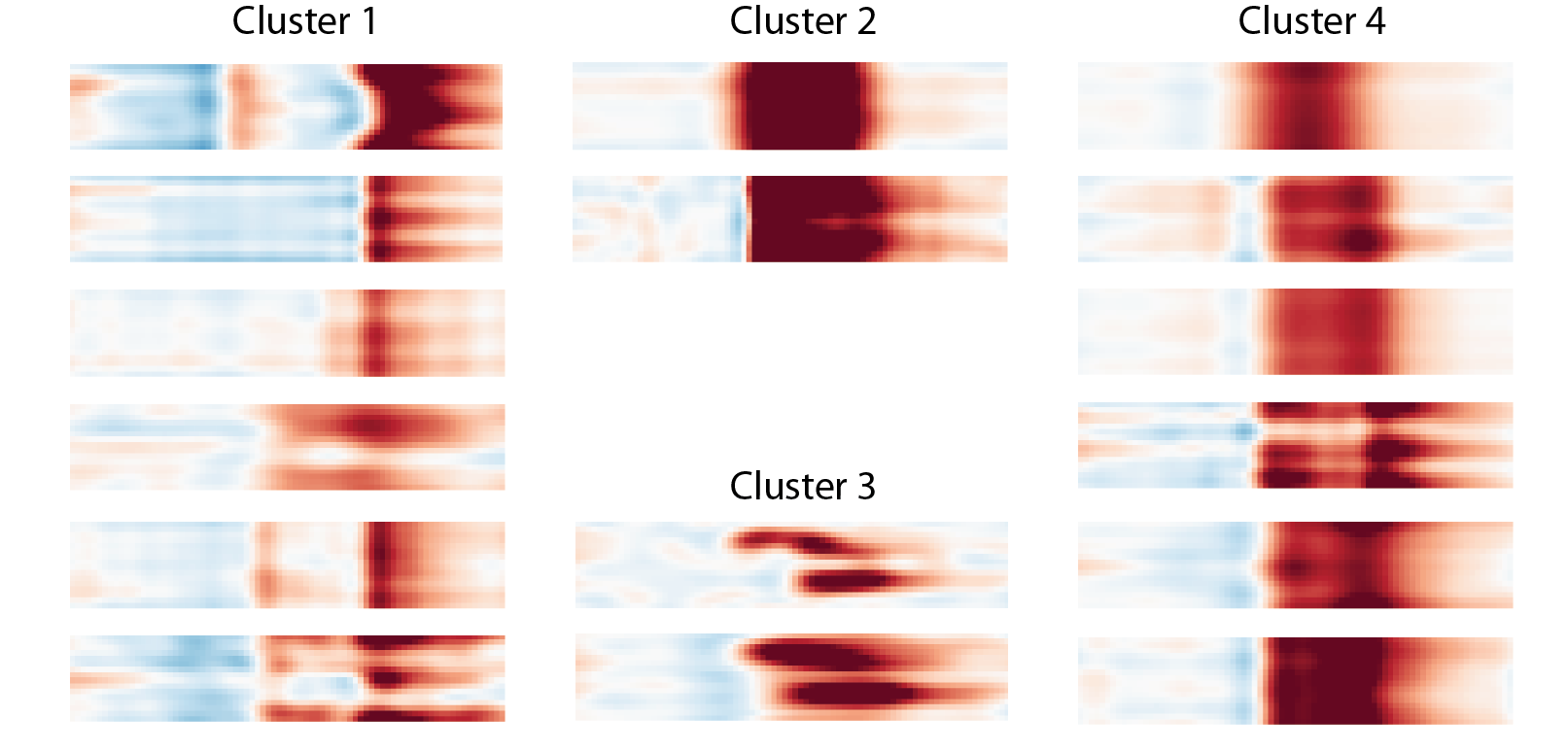

Supplement: S2 Fig — Model: Product of RBF kernel (time) with RBF kernel (direction), 300 inducing inputs, 25 iterations per fit, best of 3 fits per model. Each heat map corresponds to the posterior mean of the fitted GP. Columns correspond to clusters. (PNG) [file pcbi.1007205.s002.png]
